# Electric Railways

**Published:** 1902-01-11

**Authors:** 


### ELECTRIC RAILWAYS.

THE recent terrible accident upon the Liverpool Electric Railway, and the various expressions of opinion to which it has given rise, are sufficient to show that the methods of working and the general surroundings of such railways are matters which should receive the prompt and earnest consideration of Parliament; and that, in any conditions or restrictions which may hereafter be imposed upon them, security should be taken for the due fulfilment of the sanitary requirements of the case. We all know with how much reason the present generation may blame the want of foresight displayed by previous ones with regard to many questions of public concern, and especially with regard to matters of universal requirement, such as the supply of gas, of water, and of conveyances, and we may not unreasonably ask from our rulers that this last and greatest of them all, at a time when the means for its fulfilment are evidently on the eve of a new departure, should not be suffered to fall into the uncontrolled hands of gangs of company promoters, indifferent to every other consideration than that of the profits to be made out of the particular line or system which it is their interest to advance. It is obvious that Parliament should be in a position to lay down general principles to which all new schemes for metropolitan or suburban railways or tramways should be made to conform in such a manner as to render them parts of an uniform and well-considered whole; and that, among the several conditions to be insisted upon as preliminaries to the grant of powers, a prominent place should be held by those which provide, as far as provision may be possible, for the maintenance of all precautions necessary for the health and safety of the passengers.

Soon after the occurrence of the Liverpool accident, and in an article referring to it, the *Times* paper commented upon the management of the South London Electric Railway, with reference to the overcrowding of its morning and evening trains; and the justice of the comments in question was speedily shown by letters from long-suffering correspondents. The article also called forth a letter of feeble protest from the Chairman of the company, couched in terms of what lawyers call "confession and avoidance," and practically resting his defence upon the plea that the passengers were there, and that they must be carried somehow. He admitted, in fact, that the morning and evening demands upon the line were in excess of its capacity. The ordinary carriage is but little wider than an omnibus, is partially divided into two compartments, each seating eight persons on each side, but with an open gangway running its whole length, and is high enough for an adult man to stand erect. When travelling the doors at each end are shut, and the arrangements for ventilation are not conspicuous, while the air in the tunnel, which is carried to and fro by the trains and is very manifest as what people call a "draught," is nevertheless excessively foul. When the line has ceased working for the night, anyone who passes a station will obtain a whiff of this air as it slowly ascends, and will be reminded of the sensation experienced on passing from out of doors into an unventilated and overcrowded schoolroom, in which the pupils are drawn from a class by which personal cleanliness is not carried to excess. When 32 people are seated in such a carriage, the air-supply is deficient in quantity and bad in quality, but when, in addition to the 32, the gangway is occupied by 16 more who are standing up, the passengers are made fully acquainted with the sensations preliminary to suffocation.

We need not contemplate a catastrophe so horrible as that of Liverpool, against which, according to its Chairman, the line is secure; but it requires no great degree of imagination to realise the possibility of an accidental detention, which, indeed, has more than once occurred, but which, with a proper number of passengers, all seated at the same level, might be no more than a temporary inconvenience. What would it be with 48 passengers instead of 32, a large proportion of them women, especially if darkness were a complication of the scene. Many of the women would faint, some at least of the men would struggle and fight for the positions in which they fancied the best air might be attainable. The Black Hole of Calcutta would be repeated on a small scale. Such an event, it may well be hoped, will not occur; but even if it should not, the atmospheric conditions of the line are such as the law ought not to tolerate.

